# The microbiome of oral leukoplakia shows enrichment in *Fusobacteria* and *Rothia* species

**DOI:** 10.1080/20002297.2017.1325253

**Published:** 2017-06-01

**Authors:** Abdrazak Amer, Sheila Galvin, Claire Healy, Gary P. Moran

**Affiliations:** ^a^ Trinity College, University of Dublin, Dublin, Ireland

## Abstract

The current study was carried out to determine if changes in the oral microbiome were associated with oral leukoplakia. Swabs of oral leukoplakias were taken from 36 patients. Contralateral normal tissue was also swabbed. Swabs from 35 control patients without symptoms of leukoplakia were also taken. DNA was extracted and the V1V2 region of the 16s rRNA gene was sequenced using the Illumina MiSeq and analysed using the Mothur software package.

The structure of oral mucosal communities was most affected by smoking and the location of the site (AMOVA p < 0.01). Analysis of the constituents of these communities using LEfSe showed that *Fusobacterium sp*. and *Leptotrichia sp*. were enriched on leukoplakia sites. Patients with leukoplakia also showed enrichment for *Rothia mucilaginosa* and *Campylobacter sp*. Quantitative RT-PCR also showed that leukoplakias from lingual sites were more likely to be colonised by *Candida sp*. Analysis of these enrichments identified specific co-localisation patterns (Pearson correlation P <0.01) including *Leptotrichia sp., Prevotella sp*. and *Campylobacter concisus; F. nucleatum, Alloprevotella tannerae* and *C. gracilis*, amongst others.

*Fusobacteria* have been implicated in the progression of colorectal carcinoma and further studies are now required to determine if these microorganisms are linked to the development of OSCC.

